# Antifungal Soybean Protein Concentrate Adhesive as Binder of Rice Husk Particleboards

**DOI:** 10.3390/polym13203540

**Published:** 2021-10-14

**Authors:** Andrés Larregle, Mayra Chalapud, Florencia Fangio, Emiliano M. Ciannamea, Pablo M. Stefani, Josefa F. Martucci, Roxana A. Ruseckaite

**Affiliations:** 1 Hospital Interzonal Especializado Materno Infantil (HIEMI), Don V. Tetamanti, Castelli 2450, Mar del Plata 7600, Argentina; andreslarregle@gmail.com; 2 Instituto de Investigaciones en Ciencia y Tecnología de Materiales (INTEMA), Consejo Nacional de Investigaciones Científicas y Técnicas (CONICET), Universidad Nacional de Mar del Plata (UNMdP), Av. Colón 10850, Mar del Plata 7600, Argentina; mchalapud@fi.mdp.edu.ar (M.C.); emiliano@fi.mdp.edu.ar (E.M.C.); jmartucci@fi.mdp.edu.ar (J.F.M.); 3 Departamento de Química y Bioquímica, Facultad de Ciencia Exactas y Naturales, Universidad Nacional de mar del Pla-ta (UNMdP), Dean Funes 3350, Mar del Plata 7600, Argentina; mfangio@gmail.com

**Keywords:** carvacrol, soybean protein adhesive, mold resistance, particleboard performance

## Abstract

The aim of this research was to prepare an antifungal soybean protein concentrate (SPC) adhesive containing carvacrol (CRV) as a bioactive agent able to delay the attack of molds and yeast during storage of SPC adhesive at 4 °C as water-based systems. CRV was incorporated in SPC slurry at 0.5% *v*/*v* (~10 times its minimum inhibitory concentration against *Aspergillus terreus*, used as model fungus), to ensure its long-term action. CRV scarcely altered the thermal properties, structure and apparent viscosity of SPC adhesive. Active SPC aqueous dispersion was microbiologically stable for at least 30 days at 4 °C where the colonization begins, while control SPC was visually colonized from the second day. Rice husk (RH) particleboards of density ~900 kg/m^3^ were manufactured using the active SPC stored for 0, 10, 20, and 30 days as a binder. Modulus of elasticity, modulus of rupture and internal bond of RH–control SPC (without CRV) panels were 12.3 MPa, 2.65 GPa and 0.27 MPa, respectively, and were statistically unaltered compared with those obtained with fresh SPC, regardless of the presence of CRV or the storage time. This last implies that active SPC should not necessarily have to be prepared daily and/or be used immediately after its preparation. Since it is microbiologically stabilized, it can be store at least for 30 days, ensuring the stability of the protein. The quality of the adhesive was evidenced by the consistent properties of the adhesive, expanding its potential use and commercialization.

## 1. Introduction

Synthetic adhesives such as amino-based, phenolic and isocyanate have dominated the wood composites and particleboards industry due to their low cost, low curing temperature, short pressing time, aqueous solubility, and high dry bond strength, among other important characteristics [1,2,3]. However, there are three main driving forces leading the gradual replacement of such synthetic systems with formaldehyde-free bio-sourced substitutes: the fluctuating price of the oil, the awareness over the limited fossil resources and the concern for lowering indoor air pollution associated with formaldehyde emission [4]. Formaldehyde has been classified in 2004 by the International Agency for Research of Cancer (IARC) as carcinogenic class I and more recently, in 2016 the European Union has re-categorized formaldehyde as carcinogen class 1 B [1].

Proteins (e.g., soy, whey, gelatin, blood, wheat), are examples of valuable and worldwide accessible alternative bioresources able to provide environmentally friendly adhesives [5]. Soybean proteins (SPs) have been the subject of extensive research as raw materials of wood adhesives [6,7,8,9,10,11,12,13,14,15,16,17], because they are renewably sourced, easily processed and available worldwide at reasonable cost. SP adhesives are both non-toxic and economical, but certain obstacles are associated with SP adhesives such as their poor moisture and microbiological resistance and reduced shelf life [12].

SP-based adhesives are usually applied as aqueous dispersions [7,13,18], however the shelf life of this water-based system is limited due to the potential hydrolysis, aggregation and susceptibility of SP proteins to microbial attack [12,16,19]. Therefore, the challenge remains to obtain SP-based adhesives with high water and microbial resistance to expand their potential application. Several studies have addressed this limitation. Khosravi et al. (2010), stored soybean protein isolate (SPI) and wheat gluten (WG) dispersions up to 4 days at room temperature using small amount of commercially available antimicrobials [20]. Particleboards produced with the dispersions stored for more than 1 day before their use, exhibited poor mechanical properties and water resistance [20]. He et al. (2016), stored cottonseed meal adhesive slurries at ambient temperature for 8 days. The change in viscosity of the adhesives was dependent on the pH of the slurries, but microbial attack was evident from day 4 [21]. Adhesives made from wheat flour and thick spent sulfite liquor (TSSL) were stored for 30 days at room temperature and humidity [22]. The wood particleboards bonded with the stored adhesive exhibited lower IB values than those obtained with freshly prepared adhesive, but still passing the standard EN 312 for type II particleboards [22]. Gu and co-workers (2020), produced a hybrid adhesive combining SP, hyperbranched polyesters (HBPEs) and sodium borate, with modulated mechanical strength and strong activity against *S. areus* [12]. Jin et al. (2020) synthesized an antibacterial adhesive based on SM, silicone, catecholamine-based tannic acid and copper ions, with antimicrobial action against *E. coli* and *S. aureus* due to the synergistic effect of tannic acid (polyphenol) and copper (II) ions [19]. Bai et al. (2020) mixed alkali-modified blood meal (mBM) with soybean meal, 1,6-hexane dioldiglycidyl ether (HDE) and zinc ions to produce anti-mildew protein adhesives, stables at least for 14 days [23]. Li et al. (2020), incorporated three kinds of preservatives namely nano-Ag/TiO2, zinc pyrithione, and 4-cumylphenol to soy meal adhesives enhanced the resistance against fungi and molds for at least 15 days with minor modifications in the viscosity and solid content of the adhesives [16]. Li et al. (2019), used freeze-drying the protein adhesive and rehydrating before use, as strategy to overcome the “wet problem”. However, the authors observed significant changes in soybean meal (SM) powder stored at ambient temperature and humidity for six months due to ageing of SM over time. Such evolution negatively affected the water resistance and shear strength of the SM adhesive-bonded three-layer plywood [14].

Our work is currently focused on the design of soybean protein concentrate (SPC) adhesives and the study of their performance as binders of rice husks particleboards (RHP) of different densities [7,8,10,24,25]. We found that SPC adhesives treated with boric acid resulted in RHP with improved mechanical properties and water resistance [7]. However, in our experience, SPC aqueous dispersions can be subject to microbial deterioration under ambient conditions resulting in short shelf life, less than 1 week. Thus, our challenge is to upgrade the storage stability of SPC aqueous dispersions. In this contribution, we propose the use of carvacrol (CRV, 5-Isopropyl-2-methylphenol) natural preservative of SPC slurry during storage. Carvacrol is well-recognized by its broad-spectrum activity against spoilage bacteria, yeasts and molds associated with the position of the hydroxyl group in the aromatic ring [26,27]. In addition, it is classified as GRAS (Generally Recognized as Safe) by the US Food and Drug Administration (FDA).

In this context, the aim of this work was to examine the effect of carvacrol on the temporal stability of SPC aqueous dispersion upon storage at 4 °C for 30 days. The influence of CRV on the apparent viscosity, thermal properties and fungal stability of SPC dispersion was assessed. Also, the performance of rice husk particleboards produced with the active SPC adhesive previously stored for 10, 20, and 30 days was evaluated in terms of modulus of elasticity (MOE), modulus of rupture (MOR) and internal bond (IB).

## 2. Materials and Methods

### 2.1. Materials

Soybean protein concentrate powder (SPC, Solcom S 110) with an average particle size passing through a 100 mesh and 7% moisture, 69% protein, 1% fat, 3% fibers, 5% ash and about 15% non-starch polysaccharides (NSP, mainly cellulose, non cellulose polymers and pectin polysaccharides) as mean composition, was obtained from Cordis SA (Villa Luzuriaga, Buenos Aires, Argentina). Carvacrol (CRV, 5-Isopropyl-2-methylphenol, 98%) was provided by Sigma-Aldrich (St. Louis, MO, USA). Sodium dodecylsulphate (SDS, CH_3_(CH_2_)_11_OS(=O)_2_O^−^Na^+^, Anedra) was used as surfactant. Rice husk (RH) was supplied by a local rice miller in Entre Ríos (Argentina) and used as substitute for chipped wood in particleboard production. Boric acid (BA, H_3_BO_3_) was analytical grade and purchased from the Sigma Chemical Co. (St. Louis, MO, USA). Sabouraud dextrose agar (Britania, Buenos Aires, Argentina) and liquid medium (Britania, Buenos Aires, Argentina) were used as a mold culture medium.

### 2.2. Antifungal Strain and Inoculum and Determination of Minimum Inhibitory Concentration

The antifungal activity of pure CRV was tested by determining the minimum inhibitory concentration (MIC) against *Aspergillus terreus* as representative mold easily found in the environment [28]. The *A. terreus* strain was provided by the culture collection of the Mycology Laboratory of the Faculty of Science, Universidad Nacional de Mar del Plata (Mar del Plata, Argentina). The fungal strain was incubated in agar Sabouraud for 7 days at 35 °C [29]. To prepare the inoculum, the surface of the agar was washed with 5 mL of sterile saline (0.85%) and the suspension formed by conidia and spores was harvested, transferred to a sterile tube and allowed to stand for 3–5 min. Then, the supernatant was collected in a sterile tube and shaken with the aid of a Vortex for 15 sec. The optical density (OD) was adjusted to 0.09–0.13 by using an UV-visible spectrophotometer at 530 nm [30], which corresponded to 0.5 in McFarland scale (2 to 4 × 10^6^ UFC/mL), as verified by plate count in agar Sabouraud.

MIC was determined by the microdilution method (CLSI 2002). For it, 100 µL of Sabouraud liquid medium (Sabouraud 2X) with the CRV-containing dilutions (0.4, 0.2, 0.1, 0.05, 0.025% *v*/*v*, in the presence and absence of 0.25% *v*/*v* SDS) were placed in each of the 96 well microtiter plates with 100 μL of *A. terreus* inoculum. The negative controls used 100 µL of Sabouraud 2X + 100 µL of sterile saline solution and 100 µL of 0.4% CRV dilution in distilled water + 100 µL of sterile saline solution. Positive control was 100 µL of Sabouraud 2X + 100 µL of the inoculum. The micro titer plates were capped and incubated at 35 °C for 7 d. The lowest concentration of these drugs capable of inhibiting fungal growth by 100% as observed in the wells was determined as the MIC value. Each group was made by triplicate and the results were reported as the mean value.

### 2.3. Preparation of the Active Adhesive

Boric acid-treated SPC (control, SPCc) adhesive was prepared as described elsewhere [5]. Typically, SPC was dispersed in 3 wt.% H_3_BO_3_ solution (BA) at SPC: BA solution ratio 1:10 under stirring (500 rpm) at room temperature for 2 h. SPCc dispersion showed a pH of about 6 and the total solid content was 10 wt.%. Active adhesive (SPCa) was obtained by dispersing CRV (0.5% *v*/*v*) and SDS (0.25% *v*/*v*) into the SPCc slurry. The system was homogenized at 20,000 rpm (Ultraturax T 25 basic, IKA-Werke GMBH & Co., Staufen im Breisgau, Germany) for 5 min at ambient temperature. Afterwards, the mixture was stirred for 2 h at 500 rpm at ambient temperature. All adhesives were portioned, bottled in plastic containers and stored at 4 °C for 30 days. An individual SPCa bottle was taken from the fridge at 0, 10, 20 and 30 days to prepare the particleboards. The active adhesive retrieved at different storage time was named as SPCax, where X identifies the storage time in days.

### 2.4. Fungal Stability of Soybean Protein Concentrate (SPC) Adhesives during Storage

SPCc and SPCa adhesives were tested every day over the storage period, from day 0 (the day of the preparation and storage) up to day 30. The adhesives were taken out daily from the refrigerator and magnetically stirred at 500 rpm until reaching ambient temperature. An aliquot of each adhesive was aseptically extracted and cultured in agar Sabouraud for 7 d at 35 °C. The plates were observed qualitatively for fungal growth and changes were recorded with a digital camera

### 2.5. Adhesive Characterization

#### 2.5.1. Viscosity

The apparent viscosity of the freshly prepared SPC-based adhesives (day 0) was measured with a Brookfield DV-III plate and cone viscometer (Middleboro, USA) at room temperature. The adhesive samples were prepared and transferred into the sample holder of the viscometer. All viscosity measurements were recorded against the shear rate. Reported values were the average of three measurements.

#### 2.5.2. Differential Scanning Calorimetry (DSC)

DSC thermograms of freshly prepared adhesives were obtained using a DSC-50 Perkin-Elmer (Waltham, MA, USA) under nitrogen atmosphere (20 mL/min). For each sample about 5 mg were weighed in closed aluminum pans and then submitted to a temperature scanning from ambient up to 200 °C at a heating rate of 10 °C·min^−1^.

#### 2.5.3. Attenuated Total Reflectance–Fourier Transformed Infrared Spectroscopy (ATR-FTIR)

ATR-FTIR spectra of freshly prepared adhesives were recorded on a Thermo Fisher Scientific Nicolet 6700 spectrometer (Madison, WI, USA). All runs were performed between 400 and 4000 cm^−1^ using an attenuated total reflection (ATR) accessory with a diamond ATR crystal using 32 scans with 4 cm^−1.^

#### 2.5.4. Thermogravimetric Analysis TGA

Non-isothermal TGA measurements of freshly prepared adhesives were carried out using a thermogravimetric analyzer TGA-50 Shimadzu (Shimadzu Corp., Tokio, Japan), in the range of 25–900 °C at 10 °C·min^−1^ and under air atmosphere (flow rate 200 mL min^−1^). The sample weight in all tests was approximately 7–10 mg using platinum crucibles.

#### 2.5.5. Making of Particleboards

Particleboards were prepared according to our previous works [7,8,10]. RH was repeatedly washed with distilled water and dried until constant weight in an air-circulating oven at 80 ± 2 °C (equilibrium moisture content 8%). SPC-based active adhesives were removed from the refrigerator at different storage times (0, 10, 20, and 30 days), allowed to reach ambient temperature, stirred for 20 min and then used in particleboard manufacture. The appropriate amount of RH and SPC-based adhesives were blended in an orbital mixer (M.B.Z., San Justo, Buenos Aires, Argentina) at room temperature for 10 min, and then dried in an oven at 80 °C until the mixture reached 40% of moisture. The amount of resinated mixture was placed in a stainless-steel mold (25 × 25 cm^2^) at room temperature. The mass of mixture was adjusted to produce particleboards with density about 900 kg/m^3^. Pressure and temperature were applied (2.9 MPa, 140 °C) for 25 min reaching a final thickness of 4.5 mm. Particleboards were named as RH-SPCc for control adhesive and RH-SPCax, for boards bonded with the active adhesive where x means the storage time in days. Two boards for each adhesive were used for testing. All panels were conditioned in an environmental chamber at 65% relative humidity and 25 ± °C until constant weight before testing.

### 2.6. Physical and Mechanical Testing

Mean thickness was measured using a digital micrometer (Asimeto model IP65, Weißbach, Germany, accuracy 0–25 ± 0.01 mm) at five random locations (center and perimeter) of each specimen and averaged. Density (D), moisture content (MC), and the mechanical properties including internal bond (IB), modulus of rupture (MOR) and modulus of elasticity (MOE) were carried out according to the standard method ASTM D 1037 12 [31]. Density was measured gravimetrically using an analytical balance (Ohaus, Parsippany, NJ, USA, accuracy ±0.0001), and determined on samples with dimensions of 50 mm × 50 mm × thickness (mm). Six samples of each kind of adhesive-RH particleboard were analyzed. The same samples were dried at 105 °C for 24 h and MC (%) was determined. Mechanical properties were assessed using a universal test machine (Instron 4467, Buckinghamshire, England). Boards were sawed into pieces with dimensions of 50 mm × 200 mm × thickness (mm). MOE and MOR were tested at a crosshead speed of 2.9 mm/min and 140 mm of spam. Results were the average of six determinations per adhesive formulation. IB was obtained at a crosshead speed of 1.33 mm/min on samples with dimensions 50 mm × 50 mm × thickness (mm). Reported results were the average of eight measurements for each kind of board.

The average values observed for the physical and mechanical properties were compared with the minimum required by the regulation of the American National Standards Institute ANSI A208.1-2016 that sets forth requirements and test methods for dimensional tolerances, physical and mechanical properties and formaldehyde emissions for particleboards [32].

### 2.7. Statistic

Mechanical and physical data were analyzed statistically. Significant differences among average means of treatments were analyzed performing ANOVA and Tukey’s test (α = 0.05), using the statistical analysis software OriginPro version 8.5.0.

## 3. Results and Discussion

### 3.1. Selection of the Active Adhesive Formulation

The inclusion of CRV in the formulation must confer microbiological stability to the adhesive without negatively affecting the adhesive’s performance. Since CRV is inherently hydrophobic (HLB ~4.15), sodium dodecylsulphate (SDS, an anionic detergent) was added to the aqueous SPC slurry to get stable dispersions. SDS consists of a polar sulfate head that strongly binds to the positively charged protein groups, and a hydrophobic 12-carbon chain that interacts with hydrophobic regions of proteins [33], keeping them in solution, and facilitating the interaction with hydrophobic additives [12,17]. SDS is also reported to interact with denatured proteins, suggesting that interactions are independent of the conformation, charge and ionization state [32]. The amount of SDS to be included in SPC dispersion was fixed at 0.25% *v*/*v* based on the results of exploratory studies about the influence of SDS level on the thermal properties and apparent viscosity of SPC adhesive.

The dosage of CRV to be included in the adhesive formulation was fixed from MIC assays of pure CRV against *A. terreus*. After 7 days of incubation at 25 °C, the *A. terreus* was completely inhibited at CRV ≥ 978 ppm in the absence of SDS, and CRV > 485 ppm in the presence of 0.25% *v*/*v* SDS. The positive control containing medium and inoculum evidenced growth in all wells of the microdilution plate whereas no growth was detected in the sterile control. The improved effectiveness of CRV in the presence of SDS is due to a better water-solubility and stability of the active adhesive that favors its permeability and solubility in the phospholipid membrane of the microorganism causing structural and functional damage, as observed for eugenol-SDS emulsions [34]. The MIC values obtained in this work were significantly higher than those reported by Sokovic et al. (2002) [26], who found an inhibitory effect of CRV against *A. terreus* at 0.1 ± 0.01 to 0.2 ± 0.016 µL/mL (about 98–196 ppm). Such discrepancies with previous reports could be ascribed to differences in the strain and assay conditions. From MIC results, the dose of CRV to be included in SPC formulation was arbitrarily fixed in 0.5% *v*/*v* (about 10 times the MIC value), to keep CRV concentration higher than the MIC during the whole storage period since CRV loss is possible due to volatilization during thermo-compression [27].

### 3.2. Characterization of the Active SPC Adhesive

**Figure 1:**
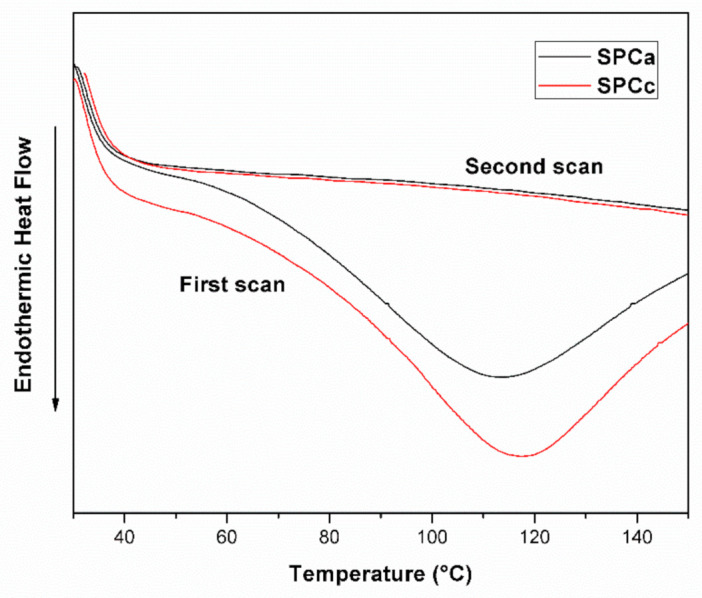
Differential scanning calorimetry (DSC) thermograms of SPCc (soybean protein concentrate) and SPCa (heating rate 10 °C·min^−1^, under nitrogen atmosphere).

The unfolding step in proteins is usually a highly cooperative transition accompanied by a significant uptake of heat, which is revealed as endothermic peaks in the DSC thermogram [13]. DSC curves of raw SPC powder and lyophilized SPCc and SPCa are shown in Figure 1. SPC powder as well as both SPC-based adhesives exhibited a broad endothermic band in the range of 70–120 °C, peaking at 118 °C and 112 °C for SPCc and SPCa, respectively.

Endothermic peaks from 0 to 180 °C observed for soy proteins [7,17], has been attributed to the loss of residual water or hydrogen bond disruption within the protein molecules. The slight shift toward a lower temperature in SPCa was related to the hydrophobic character of CRV that reduced hydrogen bond associations between protein and water, releasing moisture earlier upon heating. After quenching to ambient temperature and reheating up to 200 °C, no transitions were detected in either of the samples (Figure 1), suggesting that protein fraction in SPC and SPC-adhesives can be considered almost completely denatured [7,17]. This comes from the industrial method of SPC production where proteins of defatted soy meal are first rendered insoluble by thermal denaturation, using humid heat (T > 90 °C), ensuring the denaturation of glycinin and β-conglycinin), and then, the heat-treated meal is extracted with hot water to dissolve the sugars [35]. As a result, the incorporation of SDS and CRV did not induce additional structural changes to soy protein.

**Figure 2:**
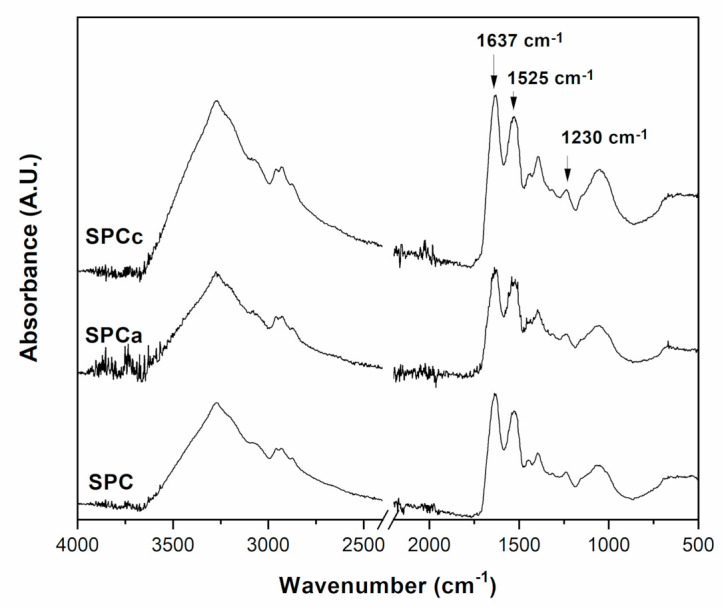
Attenuated total reflectance-Fourier transformed infrared spectroscopy (ATR-FTIR) spectra of SPC, SPCc, and SPCa.

The ATR-FTIR spectroscopy results of the SPCc and SPCa is shown in Figure 2. Both adhesives showed similar spectral patterns. The SPC spectrum was characterized by peaks of relevance at 1637 cm^−1^, 1525–1529 cm^−1^, 1230 cm^−1^ and 2354–2360 cm^−1^, assigned to amide I, (C=O stretching), amide II (N–H bending), amide III (C–N and N–H stretching) and amide A, (free and bond OH and NH groups) [7,11,12]. It is important to note that this is an SPC sample, therefore comprising many proteins, although the predominant proteins are β-conglycinin (7S) and glycinin (11S); the FTIR spectrum recorded is therefore the average absorbance of the protein concentrate. No significant differences between the spectra of SPC (without boric acid) and SPCc (SPC with boric acid). The characteristic peaks at 1440 cm^−1^ and 1130 cm^−1^ corresponding to the bonds B–O and B–O–C stretching vibrations [36], which are usually use to prove the crosslinking or complexation reaction of boric acid with hydroxyl groups, in the present case, from side-chain groups in the protein fraction and/or carbohydrate fraction of SPC [6], were not visible in the spectrum of the modified adhesives (Figure 2). This is due to the low amount of boric acid incorporated in the formulations as well as the presence of intense SPC bands in this wavenumber region. The ATR-FTIR of SPCa was similar to that of SPCc, suggesting that protein structure was preserved after modification, in accordance with DSC results. To identify the main components that build the amide I band in SPC adhesives, the second derivative [7] was applied to the 1700–1600 cm^−1^ region of the SPCc and SPCa spectra (results not shown).

Results revealed that the amide I band was composed by the superposition of at least 6 absorption bands related to different secondary structures: two bands at 1626 and 1683 cm^−1^ are associated with the amide groups involved in extended beta sheets, while that at 1651 cm^−1^ is related to α-helix and at 1643 cm^−1^ to random structures. The small peak at 1693 cm^−1^ is due to β-turns while that at 1619 cm^−1^ could be interpreted as intermolecular β-sheet structures related to protein aggregation [36]. This analysis provides evidence that SPC-based adhesives still preserve secondary structures that play a key role in the gluing ability of soy protein adhesives. The presence of CRV could not be detected by ATR-FTIR because the main peaks at 3387, 2800–2961, 1620–1458, 1359 cm^−1^ ascribed to O–H stretching, C–H *sp*^2^ stretching, C–H *sp*^3^ stretching, in-ring C–C stretching, and asymmetric and symmetric stretching vibrations of CH_3_, respectively [37,38], were hidden by those of the polypeptide structure.

**Figure 3:**
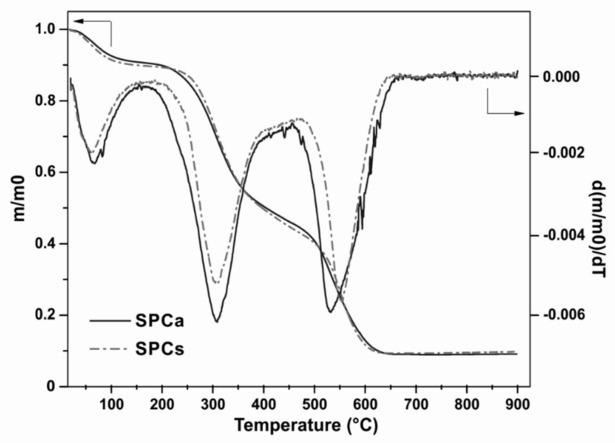
Dynamic thermogravimetric and differential thermal analysis normalized mass loss and first derivative curves of SPCc and SPCa (heating rate 10 °C·min^−1^, synthetic air atmosphere).

Thermal stability of modified-SPC adhesives was evaluated in air atmosphere that is more representative of the actual use and processing conditions. Normalized mass loss and first derivative curves of control and active SPC adhesives are shown in Figure 3. Both adhesives were stable below 300 °C, followed by two-stage decomposition process as evidenced in Figure 3. The first degradation step developed below 130 °C was indicative of the volatilization of retained moisture after lyophilization [7] and accounted for about 8–10% of the mass loss. From this temperature onwards, the rate of mass loss became rapidly significant above 200 °C and both adhesives decomposed in two steps up to about 600 °C. The second and main stage was ascribed to the degradation of soy protein, involving the scission of intermolecular and intramolecular hydrogen bonds, electrostatic interactions and random cleavage of peptide bond in protein backbone and accounted for 46% of the mass loss (Figure 3). The temperature of the maximum decomposition rate (Tmax) varied in a narrow range, i.e., 276 and 277 °C for, SPCa and SPCc. The third stage occurred above 400 °C and corresponded to the oxidative degradation of carbonaceous residues formed during the main stage, in which under synthetic air atmosphere, complete oxidation occurs. TGA results show that the most significant mass loss of both SPC-based adhesives happens above 200 °C, consequently, the adhesives will be stable at 140 °C that is the chosen pressing temperature based on our previous works [7,8,10].

**Figure 4:**
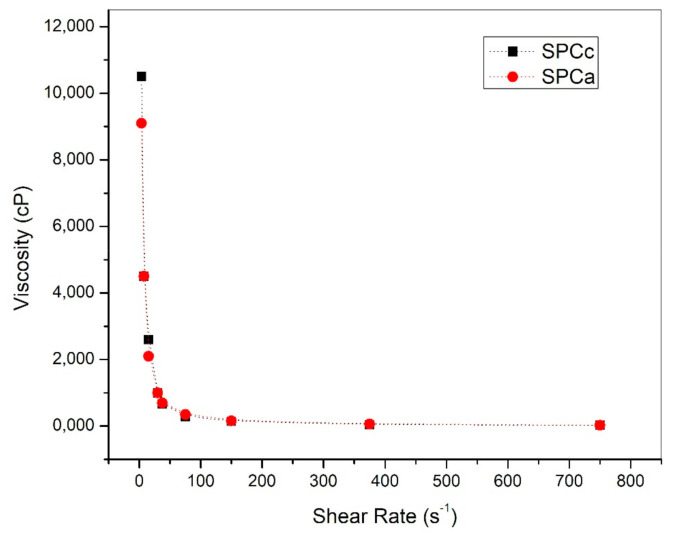
Effect of shear rate on the apparent viscosity of freshly prepared SPCc and SCPa adhesives.

The viscosity of an adhesive is a crucial property that governs its performance [11,21]. A high viscosity soybean protein adhesive, difficult the spreading and distribution onto the substrate surface (particles or veneers), limiting the gluing ability, resulting in poor bonding strength. Conversely, low viscosity adhesives tend to penetrate deep into the substrate surface, so that little adhesive is left for bonding, also resulting in low bonding strength [7,11]. Typical apparent viscosity profiles are displayed in Figure 4. All adhesives are characterized by a decrease in viscosity as a function of the shear rate (shear-thinning behavior) in accordance with previous reports for other protein-based adhesives including soybean [7,13,17] and cotton meal [21]. Shear-thinning performance of protein dispersions results from alignment of the polypeptide chains under shear flows, during which the interactions between the randomly oriented molecules are disrupted and new orientations of the protein molecules along shear planes with lower resistance to flows are established [39]. The addition of 0.25% *v*/*v* SDS and 0.5% *v*/*v* CRV reduced the initial apparent viscosity as compared with the control, i.e., from 10,500 to 8600 cps (measured at the lowest shear rate).

Lower viscosity reflects less intermolecular interactions and enhanced chain mobility [14]. Discrepancies with results informing increasing apparent viscosity with the addition of SDS [17], could be explained by the interplay of several factors. Firstly, the highly denatured structure of SPC in comparison with most of the reported studies based on SPI. Secondly, the low level of SDS used herein which makes it act more as a dispersant than as denaturant. Thirdly, CRV is a phenol, which can inset and disrupt protein-to-protein interactions reducing the viscosity. Initial viscosity values were in the range than those obtained in our previous studies for SPC adhesives [7] and for peanut meal adhesives [40] and meet the operating requirements for soy protein adhesives, from 500 to 60,000 cps [41].

### 3.3. Binder Stability during Storage

**Figure 5:**
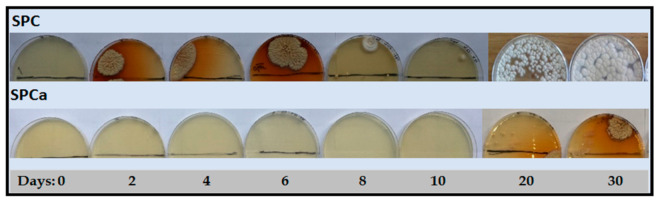
Antifungal activity of the SPCa and control SPCc in agar Sabouraud at 35 °C during 30 days.

The microbial susceptibility was qualitatively evaluated by monitoring the growth of environmental molds and yeasts over the storage period and results are summarized in Figure 5. SPCc was easily colonized by yeast from the second day. As time progressed the aspect of control SPC became moldy (Figure 5), indicating that boric acid used in the adhesive preparation (3 wt.%) has no antifungal action, at least at the concentration used. Reportedly, borate-containing SP adhesives have shown antimicrobial activity, particularly against Gram-positive *S. aureus* [12]. Authors suggested that borate containing SP adhesives have the same preservative effect than commercial BHT within 48 h. Contrarily, no colonies were observed in SPCa over 20 days (Figure 5) evidencing that CRV inhibited the fungal attack. SPCa showed enhanced shelf life, preserving the original appearance and smell.

Contrarily, SPCc was characterized by its poor microbiological stability, evidenced by the plate completely colonized by yeast and molds and an unpleasant smell due to protein degradation probably due to the action of proteolytic bacteria at the later stages of the storage period. Our qualitative results proved that SPCc was more vulnerable to microbial attack than SPCa, confirming the efficiency of CRV in upgrading the shelf life of SPC aqueous suspensions for at least 30 days at 4 °C. Zinc and nano silver-containing protein adhesives have shown increased stability against molds and yeast [16,23]. However, and to the best of our knowledge, this is the first report of the anti-mold activity of carvacrol-incorporated soybean protein-based adhesives.

### 3.4. Physical and Mechanical Properties of the Particleboards

In order to evaluate whether the properties of the adhesives remained stable during the foreseen shelf life, active SPC aqueous dispersions were stored at 4 °C for 30 days in individual bottles containing the necessary amount of adhesive to elaborate the panels. Storage time of SPCa had no effect on average thickness, moisture content and density values (~900 Kg/m^3^) of the obtained particleboards (Table 1). Similarly, MOE and MOR values remained statistically invariant (*p* > 0.05) when increasing the storage time of the adhesive (Table 2), and in the same range as those produced with SPCc (at time zero, because of the inherent biological instability of SPC slurry). Therefore, neither the addition of CRV not the storage time of the adhesive aqueous dispersions modify the mechanical performance of the panels produced. Similar MOE was previously reported for high-density RH-SPCc boards [24] but MOR was slightly below the informed values.

Internal bond (IB) values were not sensitive to adhesive’s age (Table 2). No significant differences between IB values of control and active SPC, suggesting that the addition of CRV in the formulation hardly influenced the performance of the adhesive (Table 2). CRV seemingly did not interfere in the formation of chemical bonds between boric acid-modified SPC with exposed hydroxyl groups of the amorphous region of cellulose in RH during hot pressing which enhance the bonding strength. [6,7]. The low IB values found could be due to the more compact inner structure of the high-density RH boards that compromised the homogeneous distribution of the adhesive. Hussein et al. (2019), obtained IB values varying from 0.020 to 0.008 MPa for rice straw-UF particleboards with density values between 300 and 700 kg/m^3^ [42]. The authors assumed that such low IB could be a consequence of poor adsorption of the adhesive by the rice straw due to the content of silica and waxes [42]. Similar assumption can be applied to RH-SPC particleboards produced in the present work since RH was just washed with water before use. Reportedly, RH must be summited to chemical modifications, such as alkaline treatment and bleaching, that provoke morphological changes in the inner and outer surface of RH that upgrades the adhesion with polar adhesives [7,8].

Based on the US standard ANSI A208.1.2016 [32], MOE values met the requirements for high-density particleboards H1-grade (Table 2). Unfortunately, MOR and IB of all the panels were below the standard for high-density particleboards.

## 4. Conclusions

The results of this study indicate that CRV has potential as a natural preservative of SPC aqueous dispersions with upgraded shelf life when stored at 4 °C up to 30 days, where the colonization begins. The formaldehyde-free and active SPC aqueous dispersion can be prepared, stored under proper conditions and eventually used after re-dispersing at ambient temperature without altering the final properties of the produced boards. MOE, MOR and IB values of particleboards bonded with SPCa stored for 30 d were statistically unaltered, signifying that neither the addition of CRV nor the storage time elapsed modified the performance of RH-SPCa boards. Despite the mechanical properties of the particleboards not being sensitive to the adhesive’s age, further work is still necessary to enhance the performance of RH particleboards in order to enlarge their application area. As an example, IB can be boosted by employing a natural bioactive agent able to react with SPC through crosslinking or grafting and endow anti-fungal activity.

## Figures and Tables

**Table 1:** Density (kg/m^3^), thickness (mm) and moisture content (%) of particleboards glued with SPCa stored at 4 °C for 0, 10, 20 and 30 days. Control at time zero was included for comparing purposes.

Sample Name	Thickness (mm)	Density (kg/m^3^)	Moisture Content (%)
SPCc0	4.44 ±0.07 ^a^	890 ± 32 ^a^	7.92 ± 0.05 ^a^
SPCa0	4.42 ± 0.11 ^a^	910 ± 38 ^a^	7.89 ± 0.07 ^a^
SPCa10	4.52 ± 0.07 ^a^	903 ± 40 ^a^	7.73 ± 0.05 ^b^
SPCa20	4.42 ± 0.12 ^a^	905 ± 43 ^a^	7.72 ± 0.02 ^b^
SPCa30	4.37 ± 0.07 ^a^	887 ± 39 ^a^	7.89 ± 0.02 ^a^

Values are means +/− standard deviation. Means within a column followed by different letters are significantly different (*p* < 0.05).

**Table 2:** Modulus of rupture (MPa), modulus of elasticity (GPa) and internal bond (IB) mean values of RH particleboards glued with the active SPC after different storage time.

Board Name	MOR (MPa)	MOE (GPa)	IB (MPa)
RH-SPCc	12.31 ± 1.65 ^a^	2.65 ± 0.38 ^a^	0.27 ± 0.07 ^a^
RH-SPCa0	12.36 ±1.88 ^a^	2.41 ± 0.30 ^a^	0.25 ± 0.09 ^a^
RH-SPCa10	11.00 ± 0.56 ^a^	2.56 ± 0.25 ^a^	0.23 ± 0.05 ^a^
RH-SPCA20	13.31 ± 2.66 ^a^	2.74 ± 0.23 ^a^	0.29 ± 0.07 ^a^
RH-SPCa30	12.79 ± 1.42 ^a^	2.57 ± 0.14 ^a^	0.27 ± 0.07 ^a^
ANSI A208.1-2016 H1-grade	16.5	2.40	0.90

Values are means +/− standard deviation. Means within a column followed by different letters are significantly different (*p* < 0.05).

## Data Availability

The data presented in this study are available on request from the corresponding author.
